# Early Detection of Plant Viral Disease Using Hyperspectral Imaging and Deep Learning

**DOI:** 10.3390/s21030742

**Published:** 2021-01-22

**Authors:** Canh Nguyen, Vasit Sagan, Matthew Maimaitiyiming, Maitiniyazi Maimaitijiang, Sourav Bhadra, Misha T. Kwasniewski

**Affiliations:** 1 Geospatial Institute, Saint Louis University, Saint Louis, MO 63108, USA; canh.nguyen@slu.edu (C.N.); mason.maimaitijiang@slu.edu (M.M.); sourav.bhadra@slu.edu (S.B.); 2 Department of Earth and Atmospheric Sciences, Saint Louis University, St. Louis, MO 63108, USA; 3 Division of Food Sciences, University of Missouri, Columbia, MO 65211, USA; matt.maimaitiyiming@slu.edu (M.M.); mtk5407@psu.edu (M.T.K.); 4 Department of Food Sciences, The Pennsylvania State University, University Park, PA 16802, USA

**Keywords:** plant disease, spectral statistics, machine learning, 2D-CNN, 3D-CNN, grapevine vein-clearing virus (GVCV)

## Abstract

Early detection of grapevine viral diseases is critical for early interventions in order to prevent the disease from spreading to the entire vineyard. Hyperspectral remote sensing can potentially detect and quantify viral diseases in a nondestructive manner. This study utilized hyperspectral imagery at the plant level to identify and classify grapevines inoculated with the newly discovered DNA virus grapevine vein-clearing virus (GVCV) at the early asymptomatic stages. An experiment was set up at a test site at South Farm Research Center, Columbia, MO, USA (38.92 N, −92.28 W), with two grapevine groups, namely healthy and GVCV-infected, while other conditions were controlled. Images of each vine were captured by a SPECIM IQ 400–1000 nm hyperspectral sensor (Oulu, Finland). Hyperspectral images were calibrated and preprocessed to retain only grapevine pixels. A statistical approach was employed to discriminate two reflectance spectra patterns between healthy and GVCV vines. Disease-centric vegetation indices (VIs) were established and explored in terms of their importance to the classification power. Pixel-wise (spectral features) classification was performed in parallel with image-wise (joint spatial–spectral features) classification within a framework involving deep learning architectures and traditional machine learning. The results showed that: (1) the discriminative wavelength regions included the 900–940 nm range in the near-infrared (NIR) region in vines 30 days after sowing (DAS) and the entire visual (VIS) region of 400–700 nm in vines 90 DAS; (2) the normalized pheophytization index (NPQI), fluorescence ratio index 1 (FRI1), plant senescence reflectance index (PSRI), anthocyanin index (AntGitelson), and water stress and canopy temperature (WSCT) measures were the most discriminative indices; (3) the support vector machine (SVM) was effective in VI-wise classification with smaller feature spaces, while the RF classifier performed better in pixel-wise and image-wise classification with larger feature spaces; and (4) the automated 3D convolutional neural network (3D-CNN) feature extractor provided promising results over the 2D convolutional neural network (2D-CNN) in learning features from hyperspectral data cubes with a limited number of samples.

## 1. Introduction

Climate change, unpredictable precipitation patterns, and temperature variability are creating an optimal environment for virus breeding, survival, and transmission [1]. In this respect, reliable and accurate identification of plant disease at an early stage is necessary to address the current challenge in agriculture. The existing in situ measures conventionally consist of a processing chain, relying on visual inspections of the crop in the field for signs that are already clearly visible, followed by intensive diagnostic tests in laboratories. Plant reactions to and manifestations of the incidence of pest and disease are heterogeneous in the field [2], usually starting in a small region of foliage and spreading out to the whole field. Manual detection methods are, thus, time-consuming, demanding, and not ideal for large-scale and early intervention. Innovative farming methods with precision approaches would close the gap by identifying previsual indicators and affected areas, thereby preventing the disease from spreading.

Grapevine vein-clearing virus (GVCV) was recently discovered as the first DNA virus in grapevines. It is a new species in the *Badnavirus* genus of the *Calimorividae* family. GVCV-associated disease is considered a great threat to the sustainable growth and productivity of grapevines in the Midwest region of the U.S.A. [3]. Diagnostic symptoms can be observed on new shoots, whose margins become split and crinkled. The major and minor veins of the leaves look translucent and zig-zag internodes appear and become discernable. Infectious leaves are frequently misshaped and smaller, resulting in reduced vine size and a less dense canopy than in healthy vines. Severely affected vines have reduced cluster sizes, irregular shapes, and abnormal berry texture. Several common cultivars susceptible to GVCV are Chardonnay, Chardonel, and Vidal Blanc, while Norton and Chanbourcin are resistant and Vignoles and Traminette are tolerant (i.e., mild symptoms if infected). Polymerase chain reaction (PCR), which is a molecular test, is the only method that can confirm the incidence of GVCV on a vine [3].

Among passive remote sensing methods that measure the solar radiation reflected from objects, hyperspectral imaging (HSI) shows great potential as a noninvasive and nondestructive tool in monitoring plant biotic and abiotic stress [4]. This method captures and stores an object’s spectroscopy information in a spectral cube, which contains spatial information and hundreds of contiguous wavelengths in the third dimension. Hyperspectral images offer many opportunities for early sensing of plant diseases, providing previsual indicators via subtle changes in spectral reflectance due to absorption or reflectance. To the best our knowledge, there have been no studies investigating the defensive reaction of a grapevine to GVCV, so we assumed that once infected by the virus, the host grapevine would activate a protection mechanism similar to other species. The affected plant’s biochemical and biophysical properties start changing and produce a spectral signature that differs from those of healthy plants, which can be remotely sensed by spectral sensors. For example, in the early stages, visual symptoms are not present on infectious leaves, but instead a set of physiological variables starts adjusting, including the closure of stomata, a decrease of transpiration, a reduction of photosynthesis rate, and increases of fluorescence and heat emission [5]. The thermal properties of affected leaves are abnormally altered, mainly due to changes of the water content, which can also be detected in the early infection stages [6,7]. At later stages, the chlorophyll content in the leaves may be reduced by necrotic or chlorotic lesions and infected spots on the leaves can appear, with browning effects caused by senescence [8]. Such altered pigments can be recorded in visual (VIS) and near-infrared (NIR) regions of the spectrum. Structural properties such as the canopy density and leaf area of the infected plant can be scaled down, which also influence the NIR regions [9,10].

Intensive research on plant pathology scouting has leveraged hyperspectral imaging for early identification of pathogens and diseases at varying spatial, spectral, and temporal scales. Examples include using leaf reflectance to differentiate the signal change caused by the foliar pathogens in sugar beet [11,12,13], wheat [14,15], apple [16], barley [17,18], and tomato [19]. At the field scale, hyperspectral images are useful for early detection of toxigenic fungi on maize [20], yellow rust on wheat [21], orange rust on sugar cane [22], and tobacco disease [23]. The success of hyperspectral-based methods can also be seen in the improved discrimination of highly complex and unique soilborne disease patterns on sugar beet [24] and the enhanced classification accuracy of powdery mildew infection levels on wine grapes [25]. With recent developments in unmanned aerial vehicles (UAVs), airborne images have been utilized for weed monitoring and disease detection [26,27,28,29]. In a more narrow review of precision viticulture, [30] observed the resistance of three grapevine cultivars to *Plasmopara viticola* by using a hyperspectral sensor. Other studies [27,28] distinguished *Flavescence dorée* symptoms between two red and white grapevine cultivars, and their later paper differentiated two diseases, *Flavescence dorée* and grapevine trunk, in red vine cultivars. Other diseases were detected early using hyperspectral remote sensing of different grapevines such as leafroll [31,32], leaf stripe [29], yellowness, and Esca [33]. In short, applications of hyperspectral imaging technology can be categorized into: (i) early-stage detection of diseases and symptoms; (ii) differentiation among different diseases; (iii) separation of disease patterns; and (iv) the quantification of disease severity.

When processing hyperspectral vegetation imagery, one often encounters an imbalance in the limited availability of training samples and the high dimensionality of the imagery data, which is referred to as the Hughes phenomenon [34]. Different points of view among the remote sensing community have been proposed to solve the issue. The dominant approach is to extract only spectral information from each pixel of the image. This pixel-wise method assumes each pixel is a one-dimensional vector of reflectance spectra and is correspondingly labeled with the target. This generally entails a data dimensionality reduction as a preprocessing technique, followed by primary modeling tasks (e.g., classification). To reduce spectral data to manageable low-dimensional data, the most widely used approaches are spectral band selection and data transforms. The former approach is used to choose a discrete number of key wavelengths at various positions in the spectrum to calculate representative indices (e.g., vegetation Indices) [21,35,36]. As the band selection approach preserve as much spectral information as possible, the data transform approach utilizes a transformation to compact the data into a new optimal size. Common hyperspectral data extraction techniques applied in pathological studies included principal component analysis (PCA) [37], derivative analysis [38], wavelet techniques, and correlation plots [39]. It is noteworthy that the small training sample size available in the pixel-wise methods can be addressed but the methods concurrently neglect the spatial information [40].

Alternatively, one can process the hyperspectral imagery data at the image level to extract either only spatial representation or joint spatial–spectral information. If only spatial features are considered, for example by studying structural and morphological features, the spatial patterns among neighboring pixels in relation to the current pixel in a hyperspectral image will be extracted. Machine vision methods, such as using a 2D convolutional neural network (CNN) with a patch of *p* × *p* pixel data input, have been implemented to automatically obtain high-level spatial patterns. Extracting spatial features in tandem with spectral features has been shown to significantly improve model performance. The use of spatial–spectral features can be achieved through two approaches: (i) by extracting spatial features separately, for example by using a 1D-CNN or 2D-CNN [41,42] and combining the data from the spectral extractor, for example using a recurrent neural network (RNN) or long short-term memory (LSTM) [42,43]; and (ii) by leveraging three-dimensional patches (*p* × *p* × *b*) associated with *p* × *p* spatial neighborhood pixels and *b* spectral bands to fully exploit important discriminative patterns in the hyperspectral data cubes. Despite the advances in 3D-CNN architecture, very few studies have utilized this approach for hyperspectral sensing in plant disease scouting [44]. The constraints are linked to the small training set sizes available for hyperspectral image-wise models, whereas deep learning requires a very large amount of labeled data. In this study, we attempted to use a 3D-CNN architecture as a feature extractor to take full advantage of the joint spatial–spectral correlation information, which was then trained using traditional machine learning methods, with the aim of mitigating the data sample size issue in image-wise classification.

For classification tasks, traditional machine learning algorithms (support vector machine (SVM) and random forest (RF) classifier [45] algorithms) have received tremendous attention from remote sensing scholars due to their versatility and the fact that they do not require any assumptions regarding data distribution [26,46,47,48]. The SVM algorithm is a supervised machine learning algorithm in which the objective is to maximize the distance (margin) between the separating hyperplane (decision boundary) and the training samples (support vectors) closest to that hyperplane. The RF classifier is an ensemble method that combines different classifiers (decision tree in this study) into a meta-classifier that is more rigorous and is more generalizable compared to individual classifiers alone. With the RF classifier, the majority or plurality voting principle is used to select the class label that receives the most votes from individual decision trees. When modeling on small training samples, traditional machine learning algorithms generally achieve good results, however when compared to deep learning approaches modeled on larger datasets, the accuracy may remain unchanged or even decline [49]. For this reason, we customized image-wise deep learning networks and adopted SVM and RF approaches as classifiers to replace fully connected layers.

The overall aim of the current research was to investigate the capability of hyperspectral imagery for early detection of previsual grapevine vein-clearing virus (GVCV) infections in Chardonel grapevines. We aimed to address the main objectives as follows: (1) to discriminate reflectance spectra between healthy and GVCV grapevines at different stages of infection progression using a statistical test; (2) to perform an exploratory analysis to identify the importance of disease-centric vegetation indices; (3) to classify healthy and GVCV grapevines from three approaches, namely vegetation-index-based, pixel-based, and image-based approaches, using handcrafted and automated deep learning feature extractors and machine learning methods.

## 2. Materials and Methods

### 2.1. Study Area and Data Collection

**Figure 1:**
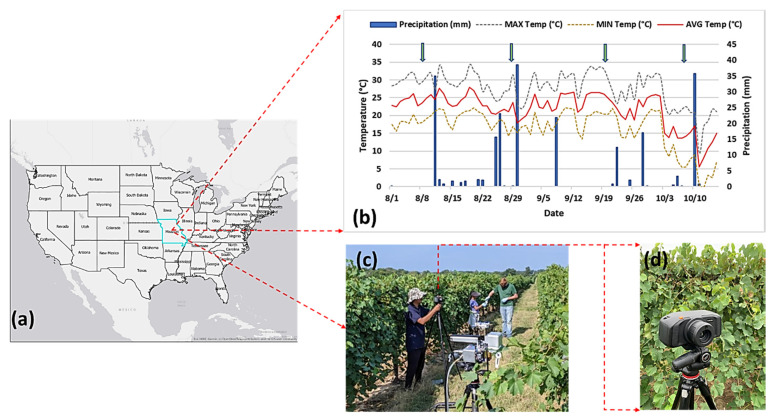
The experiment site at the University of Missouri South Farm Research Center, Columbia, MO (38.92 N, −92.28 W) (**a**). The average temperature and precipitation during the experimentation period in summer 2019 (**b**). The arrows in (**b**) indicate the hyperspectral measuring dates. The SPECIM IQ sensor with a tripod (**c**). A close-up view of the SPECIM IQ sensor (**d**). The sensor produces hyperspectral images measuring 512 × 512 pixels with 204 spectral bands.

An experiment was set up outside the University of Missouri South Farm Research Center, Columbia, MO (38.92 N, −92.28 W) (Figure 1a). As reported by an onsite weather station (http://agebb.missouri.edu/weather/stations/boone/index.htm), the average temperature and precipitation were 21.83 °C and 2.9 mm, respectively, during the experimentation period in summer 2019 (Figure 1b). Only *Chardonel* cultivar was selected for the experiment due to its susceptibility to GVCV. Vines were divided into two groups, namely the healthy group and the infected group inoculated with GVCV, as other experimental factors were controlled in similar conditions.

Hyperspectral data for healthy and infected vines were collected at different infection stages corresponding to August 7th, August 29th, September 19th, and October 8th, as summarized in Table 1. The first batch of images were acquired as nadir images when all vines were short enough to be kept in reach of the sensor tripod. Later, lateral image acquisitions were taken because the vines had already grown and were higher than the sensor tripod. There were 40 hyperspectral images in total, comprising 20 images of healthy vines and 20 images of infected vines. The hyperspectral sensor used for data collection was a SPECIM IQ sensor (Specim, Oulu, Finland), which is a portable and handheld system, as shown in Figure 1c,d. This camera can provide a measurement pipeline, performing hyperspectral capturing, data processing, and visualization. The operating hardware for the SPECIM IQ sensor utilizes push-broom technology. The incoming light passes through a prism (i.e., a convex grating) and is separated into narrow wavelengths and recorded on a light-sensitive chip. As its name implies, a push-broom device simultaneously captures a single spatial line of the image with the entire spectrum, then moves to the next line (the broom is pushed forwards). The sensor images have a static size of 512 × 512 pixels, with 204 bands ranging from 397 to 1004 nm, and with a spectral resolution of 3 nm. According to the manufacturer, the viewable area is 0.55 × 0.55 m, which can produce a spatial resolution of 1.07 mm within a distance of 1 m from the object. In this study, we captured images of vines at the canopy-level at a distances of 1–2 m. More technical specifications for the SPECIM IQ sensor can be found in [17]. As recommended for any hyperspectral system, we placed a white panel (i.e., Lambertian surface [50]) next to vines for each hyperspectral image to simultaneously record signatures of grapevines and document the characteristics of the illumination sources. The transformation from the digital number (DN) to reflectance is performed automatically based on built-in functions. SPECIM IQ has three data processing modes on board: the default recording mode (without any processing), automatic screening mode (single-class classifier), and application mode (multiple-class classifier). Both the automatic screening and application mode allow users to perform a classification process on the camera touch screen with a connection to SPECIM IQ Studio software. We selected the default recording mode to only save hyperspectral data cubes along with the RGB field of view for our further analysis.

### 2.2. Methods

#### 2.2.1. Hyperspectral Preprocessing

**Figure 2:**
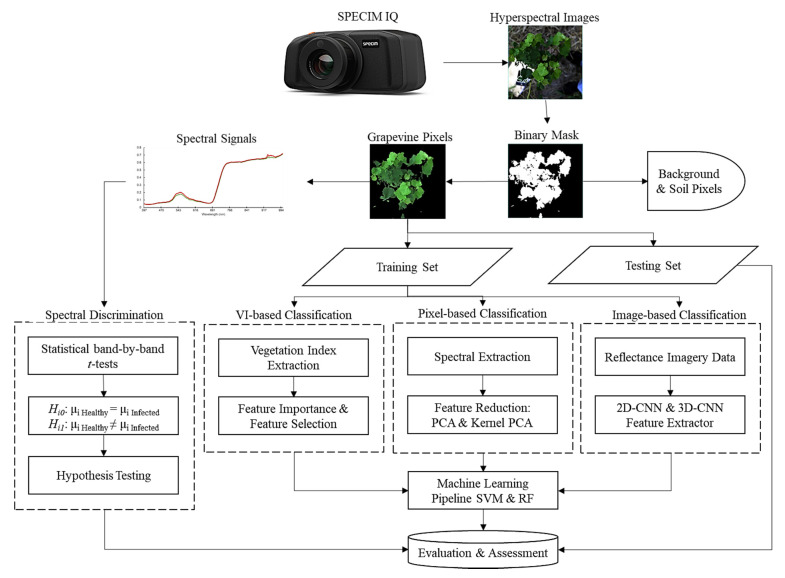
The overall workflow diagram of data processing and modeling pipeline.

**Figure 3:**
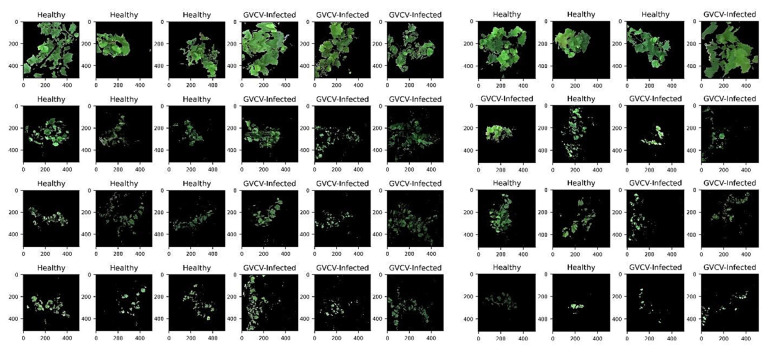
Pseudo red-green-blue (RGB) hyperspectral images in the training set (*n* = 24 images) and testing set (*n* = 16 images). The image dimensions are 512 × 512 pixels × 203 spectral bands. The row order corresponds to the measuring dates (August 7th, August 29th, September 19th, and October 8th). Training images (3 healthy and 3 GVCV vines) were randomly selected from each measure. Image backgrounds (soil, grass, sky, etc.) were masked out and only grapevines pixels were retained after the segmentation task. The August 7th images (first row) were acquired at a vertical (nadiral) angle. Subsequent images were acquired at a horizontal (lateral) angle.

The overall workflow for our research is graphically summarized in Figure 2. The data obtained from the SPECIM IQ camera were in the form of radiometrically corrected hyperspectral images, which were then imported and preliminarily examined on ENVI software (Harris Geospatial, Boulder, CO, USA). The last band was removed as noise from the hyperspectral data cubes, leaving 203 spectral bands. The ENVI built-in support vector machine (SVM) classifier with a radial basis function kernel was used to segment grapevines from the background. A binary mask layer was created to mask out the background and soil pixels, leaving only grapevine pixels (Figure 3). The overall accuracy of the SVM classification ranged from 84.4% to 97.6% across 40 hyperspectral images.

**Figure 4:**
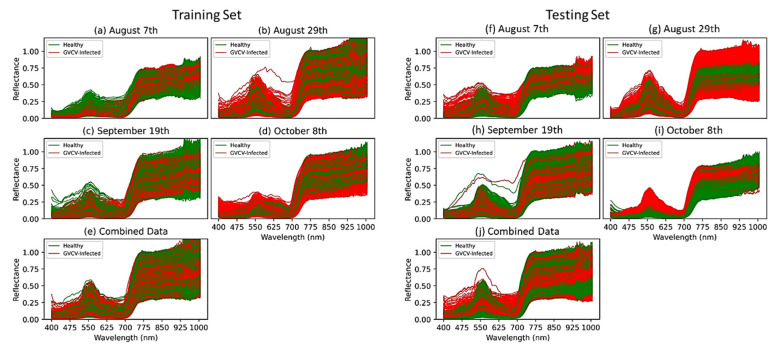
The spectral profile of 10,000 random pixels equally labeled as healthy or GVCV-infected in the training set (**a**–**e**) and testing set (**f**–**j**). Spectral data were resampled to ensure balance between the 2 classes.

For training and testing sets, we randomly selected 3 hyperspectral images from 2 classes (healthy and GVCV-infected) from each measuring date (4 dates), which generated 24 images for training and 16 images for testing. For vegetation index VI-based and pixel-based classification, pure grapevine pixels were extracted (Figure 4) and resampled to avoid class imbalance. Further, data from all dates were pooled together and named “combined data” (Figure 4e,j) with the aim of lessening the geometric sensing effects. Due to computational costs, we only trained the model with 10,000 random pixels from both classes from 24 training images (spectral signals in Figure 4a–e) and tested it on all pixels in the 16 testing images (spectral signals in Figure 4f–j).

#### 2.2.2. Reflectance Spectral Signal Discrimination

To examine the difference between the reflectance spectra of healthy and GVCV-infected vines, band-by-band independent sample *t*-tests were employed in each dataset corresponding to each collection date to explore spectral changes uniquely attributed to that infection stage and in the combined dataset. Three statistical assumptions of a *t*-test were addressed. The first assumption was that the two groups were independent of one another. The second assumption was that the dependent variable, i.e., the reflectance values of each band, was normally distributed, which was confirmed by examining the skewness (symmetry) and kurtosis (peakness) of the distribution. The third assumption was that the two groups had approximately equal variance for the dependent variable (i.e., homogeneity of variance). Levene’s tests [51] produced nonsignificant values for all bands, and thus pooled or equal variances *t*-tests were applied, as in Equation (1):

(1) $$SD_{p}^{2}=\frac{\left(n_{1}-1\right)SD_{1}^{2}+\left(n_{2}-1\right)SD_{2}^{2}}{\left(n_{1}-1\right)+\left(n_{2}-1\right)\;}$$

Equation (1) was substituted into Equation (2) to calculate the *t* values:

(2) $$=\frac{M_{1}-\;M_{2}}{SE_{(M_{1}-\;M_{2)}}}\;\;where\;\;SE_{(M_{1}-\;M_{2)}}=\;\sqrt{\frac{SD_{p}^{2}}{n_{1}}+\;\frac{SD_{p}^{2}}{n_{2}}}$$

where *M*_1_ is the reflectance mean of band *i* of healthy vines; *M*_2_ is the reflectance mean of band *i* of infected vines; *n*_1_ is the number of samples from healthy vines; *n*_2_ is the number of samples from infected vines; *SD*^2^*_p_* is the pooled variance

Because we were comparing the difference in the spectral reflectance means of each band between healthy and infected grapevines, nondirectional hypotheses were denoted:

Null hypothesis *H_i_*_0_: μ_i Healthy vine_ = μ_i GVCV vine_

Alternative hypothesis *H_i_*_1_: μ_i Healthy vine_ ≠ μ_i GVCV vine_

#### 2.2.3. Index-Wise Vegetation Classification

From grapevine pixels of hyperspectral images, spectral indices were established in relation to the state of plant properties based on previous studies in the literature review section. In Table 2, the indices were partitioned into four categories: (i) pigment, (ii) structure, (iii) physiology, and (iv) water content. Before modeling the data, a set of feature engineering techniques was achieved, including binary encoding (0: healthy; 1: infected), scaling, and feature selection. During the feature selection process, irrelevant or partially relevant vegetation indices (VIs) were filtered and removed for the purpose of reducing redundant data and algorithm complexity while improving the model accuracy and speeding up the training process. The relevance of VIs towards the output was scored by using random forest feature importance, which is also known as the mean decreased impurity (MDI) [45]. The scoring mean was then set as a threshold to retain meaningful VIs. The MDI was selected because of its robustness in avoiding the multicollinearity (high correlation among features) [35].

#### 2.2.4. Pixel-Wise Extraction and Feature Reduction

Each pixel contained 203 spectral bands. We used two different feature extraction techniques to compress and reduce the number of features, while still maintaining most of the relevant information. Principal component analysis (PCA) and kernel principal component analysis (Kernel-PCA) were both used as unsupervised data transformations. PCA can project high-dimensional data to a new subspace with fewer dimensions. PCA outputs principal components that are orthogonal axes of the new subspace and represent the directions of maximum variance. While PCA works in tandem with the assumption of the linear separability of the data, Kernel-PCA, which is an extension of PCA, can deal with nonlinear problems due to its kernelized function. Three common kernels, namely polynomial, hyperbolic tangent (sigmoid), and radial basis function (RBF), were used for hyperparameter tuning and selecting the best model.

#### 2.2.5. Machine Learning Pipeline

A sequential classification pipeline was established to preprocess and classify the 2 classes, beginning with normalizing data from 0 to 1, feature engineering (feature selection for the VI-based model and feature extraction for the pixel-based model), and the use of 2 competing classifiers (SVM and RF). To enhance the performance in dealing with highly complex and nonlinear data, SVM can be adjusted with kernels, a cost penalization function (namely *C* parameters), and gamma values. Kernel tricks can transform original data into a higher dimensional feature space where the data become separable. The *C* variable can control the penalty for misclassification and the width of the margins, and thus tune the bias–variance tradeoff. Large *C* values correspond to large error penalties and are sensitive to misclassification errors, and vice versa for smaller *C* values. Gamma values determine how quickly the class boundaries dissipate as they get further from the support vectors. In terms of RF, we considered the following hyperparameters: the number of trees in the forest, the maximum number of features considered to split a node, the maximum number of levels in each decision tree, the minimum number of data points placed in a node before the node is split, the minimum number of data points allowed in a leaf node, and whether a replacement for sampling data points (bootstrap) applied or not. The grid search technique was added to aid tuning of these hyperparameters in each step in the entire pipeline.

The metric to assess the model performance was the accuracy score calculated by all pixels and images correctly classified (true positive and false negative) over all pixels and images classified. To evaluate the models, the accuracy scores in the hold-out test set were compared. It is noteworthy that for the image-wise classification, a test set of 16 hyperspectral images (Figure 3) was withheld separately and independently from the training and validating process. For vegetation index-wise and pixel-wise classification, pure pixels from these 16 hyperspectral images (Figure 4f–j shows the hold-out test set). Further, during the training process, we employed 5-fold cross validation to lessen the probability of model underfitting and overfitting. The machine learning pipeline was built and executed on Python 3.8 and Scikit-learn library.

#### 2.2.6. Convolutional Neural Network (CNN) Feature Extractors and Image-Wise Classification

In the context of imagery data, it usually makes sense to assume the existence of spatial patterns in which adjacent pixels are more relevant than distant pixels. A convolutional layer generally performs very well on image-related tasks due to its ability to efficiently extract spatial–spectral representations of images. The architecture is typically constituted by several to hundreds of convolutional and pooling (subsampling) layers that are followed by several fully connected layers at the end. Early convolutional layers serve as feature extractors that can extract low-level features such as edges and blobs from raw images. These low-level features are combined together in a layer-wise manner to form high-level features as complex shapes such as contours. The convolutional layers usually work in tandem with pooling layers to reduce the network complexity, speed up computation, and avoid overfitting. For instance, the pooling layer with max operation (max pooling) reduces the height and width of the activation maps created by the earlier convolutional layer but retains the depth of the activation maps. Batch normalization layers are frequently placed between convolutional and pooling blocks. They are added to mitigate unstable gradient issues in a deep network and to reduce overfitting, respectively. After a series of convolutional and pooling layers, the three-dimensional activation map passes through flattening layer and is collapsed down to an array of one-dimensional vectors. Besides the functionality of the 2D-CNN, a 3D convolutional (3D-CNN) layer can simultaneously extract features from both spatial and spectral dimensions, thereby effectively capturing spatial–spectral patterns encoded in multiple adjacent pixels and wavelengths.

**Figure 5:**
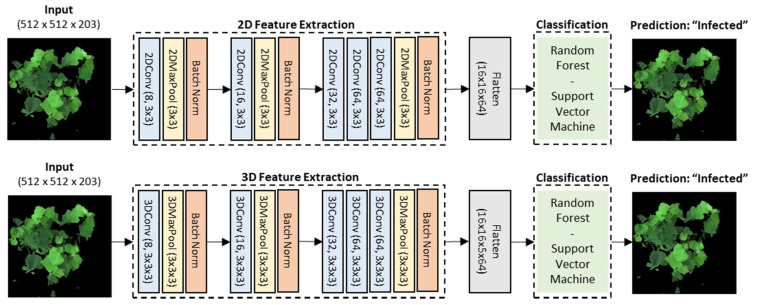
The architecture diagram for the image-wise classification. The original dimensions of the hyperspectral images were 512 × 512 pixels × 203 spectral bands. The automated feature extraction network used was an architected based on AlexNet [78], but the hyperparameters filters, kernel sizes, and pool sizes were experimented on in parallel and were kept the same for both the 2D- and 3D-CNN for a comparison. The 2D-CNN reduced the data cubes to 16 spatial features × 16 spatial features × 64 convolutional filters, while the 3D-CNN reduced the data cubes to 16 spatial features × 16 spatial features × 5 spectral features × 64 convolutional filters. Reduced features were flattened and supplied to the RF and SVM machine learning classification pipelines.

Because of the very small sample size of 40 hyperspectral images, we customized the architecture (Figure 5) by using 2D-CNN and 3D-CNN blocks as automated feature extractors and by training the reduced features on the machine learning pipeline described above. The deep learning architecture for the feature extraction was inspired from AlexNet [78] because of its simplicity and suitability for binary classification problems. Hyperparameters such as filters, kernel sizes, and pool sizes were fully investigated and selected for both 2D- and 3D-CNN models for comparison. Specifically, there were 8 convolutional filters in the first blocks, 16 and 32 in the subsequent blocks, and ending with 64 in the last layer. The kernel size was 3 × 3 pixels for the 2D-CNN model and 3 × 3 × 3 pixels for 3D-CNN with a stride of 1. The same size was also set for the max pooling layers.

## 3. Results

### 3.1. Statistical Analysis used to Discriminate between Spectral Signatures

**Figure 6:**
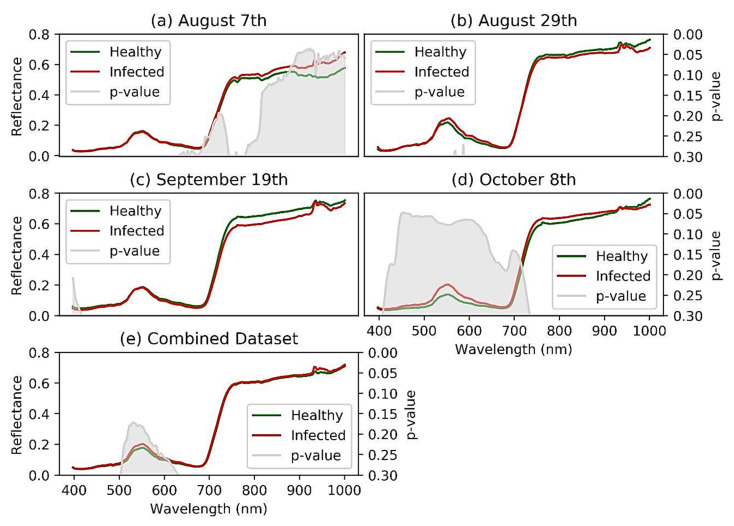
Reflectance spectra signature discrimination for healthy and GVCV-infected vines at the early infection stages. The 20-day interval hyperspectral measurements of 30 days after sowing (DAS) grapevines occurred on August 7th (**a**), August 29th (**b**), September 19th (**c**), and October 8th (**d**) in 2019. Data from all dates were pooled and named the “combined dataset”, (**e**) since all measurements were in asymptomatic stages. An independent sample *t*-test was applied to calculate *p*-values (in gray regions) so as to validate the confidence level for the spectral discrimination.

The spectra separability between healthy and GVCV-infected vines in the early infection stages was examined in Figure 6. In the earliest stage August 7th (Figure 6a), no difference was found in VIS wavelengths, but differences were found in the region of NIR wavelengths (800–1000 nm). The reflectance spectra for GVCV-infected vines were higher than the healthy ones at longer wavelengths. The *t*-test supported the difference with statistical significance in the region of 900–940 nm (*p* < 0.05). On the next measurement dates (August 29th and September 19th; Figure 6b,c, respectively), the spectra factor for the GVCV vines was slightly adjusted in the NIR region to be lower than the healthy samples. This change was small and insignificant, as the statistical evidence produced high *p*-values (*p* > 0.3) in both measurements. In the later stage on October 8th (Figure 6d), the VIS and red-edge bands (400–700 nm) were found to be most distinctive and separable between spectral values of healthy and infected vines. The healthy values were lower than the infected values, which was confirmed by a significant *t*-test in the wavelength range of 449–461 nm. The pattern for NIR wavelengths (720–920 nm) in this late asymptomatic stage was identical to the one on August 7th in that the infected signal was higher, although it was not statistically significant. When combining datasets across asymptomatic stages (Figure 6e), the discrepancy of the spectra factor for healthy and GVCV grapevines was diminished and became indistinguishable, especially in NIR wavelengths. The confidence level was also lowered to 80% (*p* < 0.2) to statistically validate the discrimination in the VIS spectra of healthy and GVCV vines. There was a prompt increase in reflectance values in the range of 925–930 nm, regardless of the dataset. This effect was caused by the atmospheric and water absorption of those bands in outside measurements under direct sunlight [17].

### 3.2. Index-Wise Classification of Vegetation

**Figure 7:**
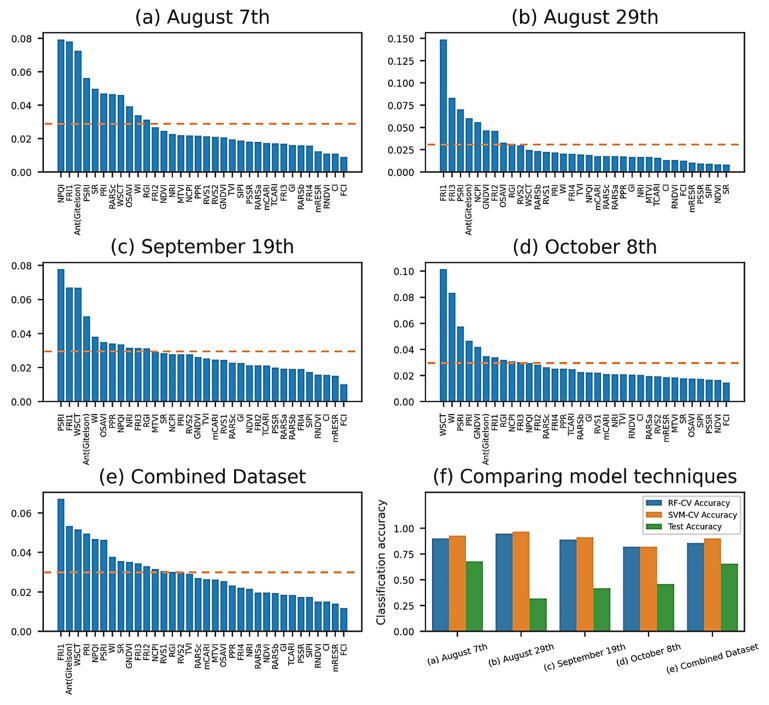
Mean decreased impurity (MDI) feature importance among datasets (**a**–**e**). The normalized scores for 34 VIs were ranked by their respective classification ability. The dashed lines indicate the mean of the scores, which was a threshold used to allow selected VIs to enter the machine learning pipeline. Vegetation-index-based classification performance (**f**). In the training process, 5-fold cross validation accuracies from SVM pipelines (orange bars) were uniformly higher than the ones from RF pipelines (blue bars), and thus SVM pipelines were selected for testing (green bars).

Generated from the random forest-based mean decreased impurity (MDI) function, the feature importance function returned the normalized scores for all 34 vegetation indices (VIs) and ranked them by their respective ability to classify healthy and GVCV-infected vines. The MDI technique utilized on August 8th (Figure 7a) suggested two physiology indices (NPQI and FRI_1_) and a pigment index (Ant_Gitelson_) as the most prominent features. For the August 29th measurements (Figure 7b), FRI_1_ became the most important index, the score for which was far higher than the others. Its importance remained for the next 20 days until September 19th (Figure 7c), in addition to another pigment index, PSRI. On this date, both water indices WSCT and WI started shifting to within the top five of the scale. On the last measurement date (Figure 7d), these water indices became the two most important features in classifying healthy and GVCV vines, whereas FRI_1_ dissipated. The feature selection technique for the combined dataset (Figure 7e) showed FRI_1_, WSCT, and Ant_Gitelson_ as the critical indices for classification power. It is worth mentioning that the sum of all normalized values equals 1.0, and thus if two or more VIs are highly correlated, one VI may have high values while the information for other VI(s) may not be fully captured. We set the mean of the scores as a threshold to allow selected features to enter a machine learning pipeline (Figure 7f) for the classification task. The SVM classified the target marginally more accurately than RF classifier, which was in turn selected for testing purposes. In the training process, the 5-fold cross-validation accuracies were as high as 96.75% for SVM classifier for the August 29th data and as low as 82.13% for the RF classifier for the October 8th data. The accuracy for the test set reached 67.81% for the August 7th data and noticeably varied for the next measuring dates. The combined data displayed better stability between training scores with 85.86% for the RF classifier, 90.24% for the SVM, and 65.70% for the testing score.

### 3.3. Pixel-Wise Classification

**Figure 8:**
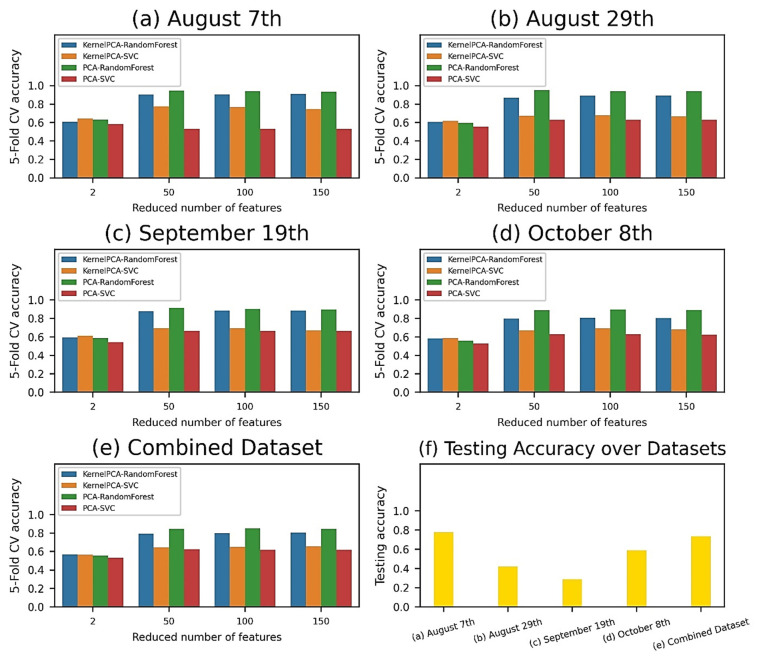
Pixel-wise classification performance for different machine learning pipelines and datasets. The 5-fold cross validation (CV) accuracy was plotted by reducing the number of features for the August 7th (**a**), August 29th (**b**), September 19th (**c**), October 8th (**d**), and combined dataset (**e**). The best pipelines were the PCA–random forest pipeline with the 50-feature model (94.70%) for August 7th data, the PCA–random forest pipeline with the 50-feature model (95.30%) for August 29th data, the PCA–random forest pipeline with the 100-feature model (91.60%) for September 19th data, the PCA–random forest pipeline with the 100-feature model (89.70%) for October 8th data, and the PCA–random forest pipeline with the 100-feature model (85.10%) for the combined dataset. These pipelines were selected for evaluation on the test set (**f**).

From 203 spectral bands of each pixel in the hyperspectral images, these features were projected to new 2-, 50-, 100-, and 150-feature spaces by using PCA and Kernel-PCA, and were subsequently supplied to a machine learning pipeline of SVM and RF classifiers (Figure 8a–e). Regardless of the datasets and classifiers, PCA and larger feature spaces (50, 100, and 150 features) worked the best and achieved over 95% validation accuracy compared to Kernel-PCA and 2 features models. RF consistently outperformed SVM for almost all datasets and feature reduction techniques. The machine learning pipelines yielded the highest cross-validation accuracy values of 94.70% (50-feature model) for the August 7th data, 95.30% (50-feature model) for the August 29th data, 91.60% (50-feature model) for the September 19th data, 89.70% (100-feature model) for the October 8th data, and 85.10% (100-feature model) for the combined dataset. These trained pipelines were thereafter evaluated on the independent test set (Figure 8f), giving accuracy scores of 77.75%, 41.89%, 28.71%, 58.80%, and 73.62% for the August 7th, August 29th, September 19th, October 8th, and combined dataset, respectively.

### 3.4. Automated 2D-CNN and 3D-CNN Feature Extraction and Image-Wise Classification

**Figure 9:**
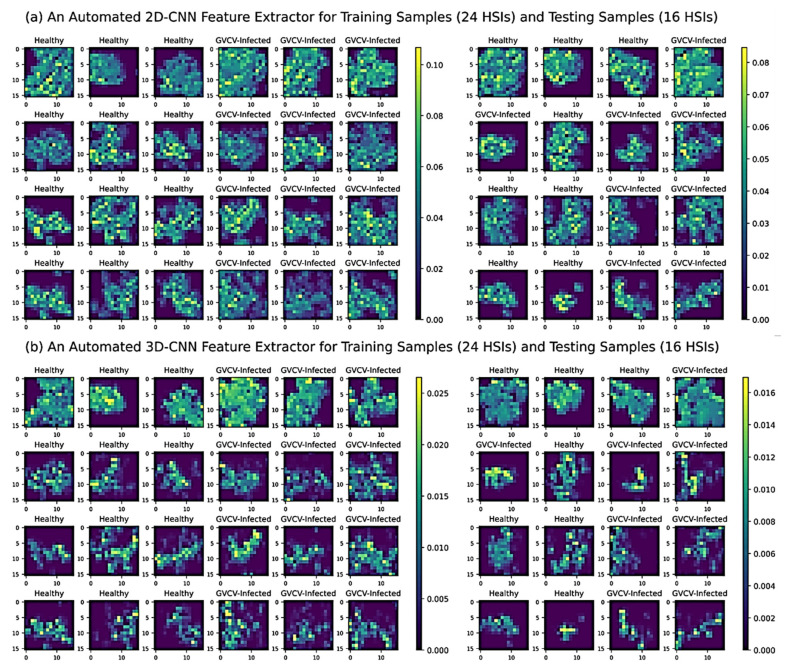
Convolutional filters and feature maps of an automated 2D-CNN feature extractor (**a**) and 3D-CNN feature extractor (**b**) for 24 training HSIs and 16 testing HSIs. From the original dimension 512 × 512 pixels × 203 bands (width × height × spectral bands), hyperspectral data cubes were reduced to a new and lower-dimensional data space of 16 × 16 pixels × 64 filters (width × height × convolutional filters) by the 2D-CNN feature extractor and 16 × 16 pixels × 5 bands × 64 filters (width × height × spectral bands × convolutional filters) by 3D-CNN feature extractor. The reduced spatial features (16 × 16) were plotted against the convolutional filter values (a random selection from 64 units). The graduated scale represents the convolutional values.

The 2D convolutional network served as an automated feature extractor to reduce the high-dimensional hyperspectral images from 512 × 512 pixels × 203 bands (width × height × spectral bands) in Figure 3 to a new and much lower dimensional data space of 16 × 16 pixels × 64 filters (width × height × convolutional filters) in Figure 9a. Similarly, the 3D-CNN feature extractors shrunk the original images to a new dimensional equivalent to the 2D-CNN’s output with 16 × 16 pixels × 5 bands × 64 filters (width × height × spectral bands × convolutional filters) in Figure 9b. The difference between the 2D-CNN and 3D-CNN feature extractors was in how the transformation of the spectral features occurred. With regard to 2D-CNN, the first convolutional layer instantly transformed all 203 spectral features and a few of the spatial features. By contrast, the 3D-CNN filters fractionally absorbed the spatial–spectral features on each layer, and as such were continually processed and reduced to 5 spectral features in the last layer. Further, with a kernel measuring 3 × 3 pixels, the 2D-CNN blocks transformed features separately in every spectral band, whereas the 3D-CNN blocks utilized a cube of 3 × 3 × 3 kernels to transform spatial–spectral features at the same time. The concurrent 3D-CNN transformation allowed it to learn features better from the hyperspectral cubes; however, it also had more trainable parameters in each block, thus making the whole model more complex, more time-consuming, and require more computational resources compared to the 2D-CNN.

**Figure 10:**
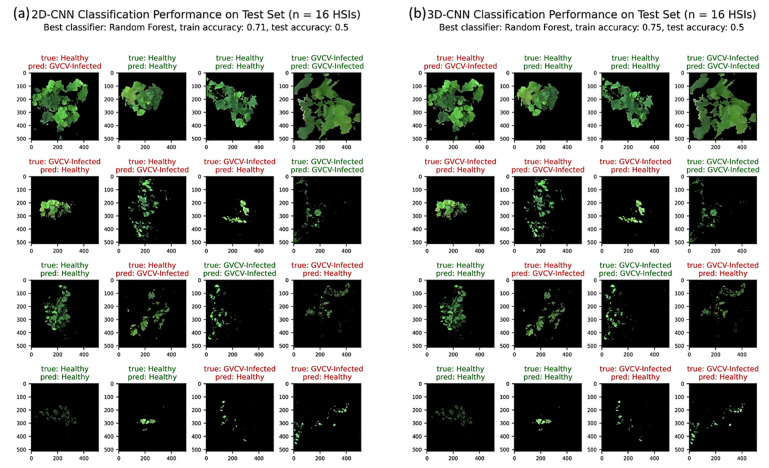
Classification performance of 2D-CNN (**a**) and 3D-CNN (**b**) models on a hold-out test set (*n* = 16 HSIs). The RF approach was the best classifier, with 71% validation accuracy for the 2D-CNN model and 75% validation accuracy for the 3D-CNN model. Both correctly classified 50% of 16 HSIs in the test set. The image subtitles indicate true and predicted classes (healthy versus GVCV-infected vines). Green image subtitles indicated a correct classification, while red subtitles indicated an incorrect classification. The row order corresponds to the measuring dates on August 7th, August 29th, September 19th, and October 8th, 2019.

In the machine learning pipeline training and validation on 24 HSIs, the random forest classifier produced the best results for both 2D-CNN and 3D-CNN models, with 71% and 75% accuracy values, respectively, for 5-fold cross validation. Figure 10a,b shows the classification performances on the test set (*n* = 16 HSIs) using 2D-CNN and 3D-CNN as automated feature extractors. The 2D-CNN testing accuracy exactly matched the accuracy of the 3D-CNN model at 50%. Both performed well on the August 7th data by correctly classifying 3 out of 4 HSIs, but not performed well on the August 29th data by falsely claiming 3 out of 4 HSIs. Overall, half of the September 19th and October 8th test images were correctly identified into their true groups as healthy or GVCV-infected vines.

## 4. Discussion

### 4.1. Reflectance Spectra Discrimination Performance

Band-wise differences in the VIS wavelength range (500–620 nm) in the combined dataset were found at a confidence level of 80% and statistical significance of *p* < 0.2. When examined in under temporal conditions, the spectral signals of GVCV vines were more discriminative at VIS wavelengths (449–461 nm, *p* < 0.1), starting at the same spectral value and progressing to significantly higher values than for the healthy group as the infection severity increased (Figure 6d). This phenomenon happened due to changing leaf pigment constituents, which dominate the VIS spectral region [79]. This supported our hypothesis and our previous research [80], stating that the pigment concentrations of disease vines will reduce, leaves will become less absorbable, and will, thus, reflect more electromagnetic energy to the sensor. Although reduced leaf pigment was observed, the vines had not yet displayed chlorosis or yellowing at those stages, as the red-edge region did not shift to lower wavelengths [73]. Considering the spectral separability in NIR wavelengths, this was noticeably discriminative between healthy and GVCV vines in the 900–940 nm wavelengths (*p* < 0.1) in the early infection stage. However, the pattern vanished in the next spectral measurements and was vaguely detected again in the late asymptomatic stage. The higher NIR spectra factor in GVCV vines reinforced our prior assumption of the plants’ physiological changes in relation to a reduction of leaf water content. Moreover, once leaves were infected, the destruction of cellular structures and the collapse of cell compactness also partially contributed to higher leaf reflectance spectra in NIR wavelengths [80]. The flipping issue that meant the NIR region values of GVCV vines were lower in August 29th and September 9th data might be due to changing the sensing angle. On August 7th, the vertical (nadir) sensing angle could capture spectroscopy images of almost all new shoots and young leaves, at which point the GVCV started. From the horizontal measurements, GVCV-affected shoots and leaves were obscured until the infectious areas spread across the whole plant in the late stage. The issue of the shooting angle is also a challenge in UAV-based disease detection, as the lower portion of the canopy cannot be captured with the nadir sensor angle. This is especially true for diseases such as the fungus *Corynespora cassicola,* which starts in the bottom part of a plant [26].

### 4.2. Interpretation of Feature Importance Analysis

With respect to the importance of the vegetation indices, the random-forest-based mean decreased impurity (MDI) feature importance was used for exploratory analysis. Among common disease-centric vegetation indices (VIs), the normalized pheophytization index (NPQI), fluorescence ratio index 1 (FRI_1_), plant senescence reflectance index (PSRI), and anthocyanin index (Ant_Gitelson_) were identified as the most discriminative indices in early stages, while the water stress and canopy temperature (WSCT) was identified as important in later stages. In more detail, the NPQI (values of 415 and 435 nm) was more sensitive to the chlorophyll degradation into pheophytin [74]. The FR_1_ (values of 630 nm and 690 nm) is a chlorophyll fluorescence indicator that is highly associated with the physiological status of photosystem II and stomatal conductance [70,72]. It is worth noting that chlorophyll fluorescence is correlated positively with photosynthesis under stress conditions and negatively under normal status [81]. PSRI is closely related to carotenoid and mesophyll cell structures [63,82], Ant_Gitelson_ (values of 550, 700, and 780 nm) is closely related to anthocyanin [52], and WSCT (values of 850 and 970 nm) is closely related to the canopy water content and temperature [77]. Previous studies also showed that the NPQI was important for early detection of *Xylella fastidiosa* (Xf)-affected olive trees [83], that FRI_1_ was important for identification of water-stressed soybean plants [84] and to estimate grapevine berry yield and quality [36], that PSRI was critically needed to characterize the spectra of peanut leaf spot disease [85], that Ant_Gitelson_ was necessary for identifying tomato spotted wilt virus (TSWV) in capsicum plants [86], and that water-related indices were essential for more accurate detection of citrus canker disease [26].

Further, in the first measure, the sensor was able to fully and stably capture the pheophytin, anthocyanin, and chlorophyll fluorescence contents that had been altered in diseased grapevines, and these images were homogeneous (Figure 3 and Figure 4), which made them successfully classifiable compared to the healthy ones (Figure 7f). In Figure 7f, the reason for the unstable and weak inference between training and testing results in the hyperspectral measurements for August 29th and September 19th data was the wide variation between FRI_1_ and PSRI values among images. It is possible that FRI_1_ chlorophyll fluorescence heavily relies on the sunlight conditions and the time of the day. One study [87] found that the magnitude of fluorescence emission and photosynthesis fluctuates the most in the afternoon, as plants are exposed to high sunlight intensity. On August 29th and September 19th, we measured the reflectance spectra at 14:00 to 15:00 (Table 1). Regarding the PSRI variation, horizontally sensing of a plant at the canopy level was only able to proportionally capture the carotenoid and mesophyll changes in the cell structure and leaves [82,88], and these proportions remarkably varied from plant to plant. Noticeably, no structural VI was found to be useful for spectral classification because of the similarity in canopy structure and color (i.e., greenness) between healthy and diseased plants in such asymptomatic periods.

### 4.3. Comparison of Classification Performances

Comparing the classification success of VI-based and pixel-based classification methods, they both achieved comparable results. Specifically, the 5-fold cross-validation accuracies ranged from 82.13% to 96.75% in the VI-based model (Figure 7f) and from 85.10% to 95.30% in the pixel-based model (Figure 8a–e). Our results also suggested that the support vector machine (SVM) was more successful with small feature data in the VI-wise classification. It indeed worked as well as the random forest (RF) classifier for the smallest 2-feature model when using pixels for classification. On the other hand, the RF classifier turned out to be the best classifier for reflectance data that had been orthogonally transformed by PCA and Kernel-PCA, which comprised a larger feature space. This clearly proved the merit of the RF classifier in modeling high-dimensional data because it intrinsically works with a random subset of features instead of all of the features of the model at each splitting point of an individual tree in the forest, thereby averaging away the feature variance. Numerous pathological and entomological vegetation studies have reported that SVM succeeded in modeling f VIs extracted from spectral bands [39,89,90], while at the same time the modeling performance of for the RF classifier was found to be stable and superlative with transformed spectral reflectance data [91,92,93].

It is hard to directly compare the classification success of the VI-based and pixel-based models to the image-based model because of the different labeled targets. Nonetheless, we maintained the same training sets, hold-out testing sets, and machine learning pipelines for all three approaches, which might provide a holistic picture of the classification problem. With the automated 2D-CNN and 3D-CNN feature extractors, the image-based machine learning pipeline gained 71% and 75% accuracy for 5-fold cross validation and 50% accuracy for testing in both networks. As we expected, the primary reason was the limitation of the small sample size (40 images) in the image-wise classification. Alternatively, whereas both VI-based and pixel-based methods mostly considered spectral features, the image-based simultaneously extracted joint spatial–spectral features. The spatial features could have been a source of noise, considering the highly fragmented portions of grapevines extracted instead of whole plants in each image (Figure 3). The foregoing feature importance analysis also indicated the usefulness of the plant structure and morphology for this classification task. All three approaches persistently classified August 29th and September 9th imagery data with limited success. It was clear that the high variation in these canopy-level datasets was caused by failure to fully capture physiological changes in leaf cells and to receive additional neighborhood electromagnetic scattering data.

## 5. Conclusions

A reliable, accurate, and nondestructive measure is critical for early identification of disease incidence in crops, thereby allowing timely intervention to prevent diseases from spreading to entire fields. This study investigated the feasibility of using hyperspectral remote sensing imagery as a nondestructive method to identify grapevines inoculated with grapevine vein-clearing virus (GVCV) in the early asymptomatic stages. With all things considered, the major conclusions included:

1. Reflectance spectra revealed useful information that was used to identify a set of optimal wavelengths to discriminate GVCV-affected vines from healthy vines in the asymptomatic stage. The discriminative wavelength regions included 900–940 nm in the NIR region in vines inoculated 30 DAS, 449–461 nm in the VIS region in vines inoculated 90 DAS, and in the entire VIS region of 400–700 nm when a lower confidence value of 90% was accepted (*p*-value of 0.1);
2. The exploratory analysis showed the importance of vegetation indices (VIs) associated with pigment, physiological, and canopy water changes. In earlier stages of GVCV infection, NPQI, FRI_1_, PSRI, and Ant_Gitelson_ were the most discriminative indices, however in the later stages WSCT was found to be important in identifying the viral disease. Correspondingly, the above indices reflected changes in the chlorophyll degradation into pheophytin, the chlorophyll fluorescence, carotenoid and mesophyll cell structures, anthocyanin levels, and canopy water and temperature statuses. Further consideration of the intensity of light illumination, sensing geometries, and measuring time must occur in order to draw conclusions regarding FRI_1_ and PSRI indices. Neither canopy structure nor greenness VIs were important in identifying GVCV disease in asymptomatic stages;
3. The classification performances of the VI-based and pixel-based models were comparable across datasets. The SVM was found to be effective in VI-wise classification with smaller feature spaces, while the RF classifier performed better in pixel-wise and image-wise classification with larger feature spaces. All classification methods were the most accurate with grapevines 30 and 90 DAS and had limited success with grapevines 50 and 70 DAS;
4. When modeling at the image level, the automated 3D-CNN feature extractor provided promising results over the 2D-CNN extractor in terms of feature learning from hyperspectral data cubes with a limited number of samples.

The findings of this study can aid viticulture farmers, wine manufacturers, and pathology and entomology scientists in early identification of the first DNA viral disease (GVCV) in grapevines in a nondestructive and efficient fashion. In addition, this study demonstrates potential frameworks for processing and modeling hyperspectral imagery data when considering only spectral features, only spatial features, and joint spatial–spectral features. The advantages of deep learning techniques were leveraged in the processing of digital images, combined with the versatility of traditional machine learning in working with a limited sample size.

## Figures and Tables

**Table 1:** Summary of sample size by grapevine health status and measurement dates. GVCV, grapevine vein-clearing virus.

	Data Measurement Time (US Central Time)	Days after Sowing (DAS)	Number of Healthy Vines	Number of GVCV-Infected Vines	Total
August 7th	10:30–11:30	30 days	6	4	10
August 29th	14:00–15:00	50 days	4	6	10
September 19th	12:00–13:00	70 days	5	5	10
October 8th	13:30–14:30	90 days	5	5	10
Total			20	20	40

**Table 2:** Extraction of vegetation indices (VIs) from hyperspectral imagery data.

No.	Vegetation Index	Acronym	Equation	References
** Pigment **				
1	Anthocyanin (Gitelson)	Ant_Gitelson_	Ant_Gitelson_ = (1/R_550_ − 1/R_700_) × R_780_	[52]
2	Chlorophyll Index	CI	CI = (R_750_ − R_705_)/(R_750_ + R_705_)	[53]
3	Optimized Soil-Adjusted Vegetation Index	OSAVI	OSAVI = (1 + 0.16) × (R_800_ − R_670_)/(R_800_ + R_670_ + 0.16)	[54]
4	Red–Green Index	RGI	RGI = R_690_/R_550_	[55]
5	Structure Intensive Pigment Index	SIPI	SIPI = (R_800_ − R_450_)/(R_800_ + R_650_)	[56]
6	Transformed Chlorophyll Absorption in Reflectance Index	TCARI	TCARI = 3 × ((R_700_ − R_670_) − 0.2 × (R_700_ − R_550_) × (R_700_/R_670_))	[57]
7	Nitrogen Reflectance Index (NRI)	NRI	NRI = (R_570_ − R_670_)/(R_570_ + R_670_)	[58]
8	Modified Chlorophyll Absorption in Reflectance Index	mCARI	mCARI = 1.2 × (2.5 × (R_761_ − R_651_) − 1.3 × (R_761_ − R_581_))	[59]
9	Photochemical Reflectance Index	PRI	PRI = (R_531_ − R_570_)/(R_531_ + R_570_)	[60]
10	Ratio Analysis of Reflectance of Spectral Chlorophyll a	RARSa	RARSa = R_675_/R_700_	[61]
11	Ratio Analysis of Reflectance of Spectral Chlorophyll b	RARSb	RARSb = R_675_/(R_700_ × R_650_)	[61]
12	Ratio Analysis of Reflectance of Spectral Chlorophyll b	RARSc	RARSc = R_760_/R_500_	[61]
13	Pigment-Specific Simple Ratio	PSSR	PSSR = R_800_/R_680_	[62]
14	Plant Senescence Reflectance Index	PSRI	PSRI = (R_660_ − R_510_)/R_760_	[63]
15	Normalized Chlorophyll Pigment Ratio Index	NCPI	NCPI = (R_670_ − R_450_)/(R_670_ + R_450_)	[56]
16	Plant Pigment Ratio	PPR	PPR = (R_550_ − R_450_)/(R_550_ + R_450_)	[64]
**Structure**				
17	Normalized Difference Vegetation Index	NDVI	NDVI = (R_860_ − R_670_)/(R_860_ + R_670_)	[65]
18	Greenness Index	GI	GI = R_554_/R_677_	[55]
19	Green NDVI	GNDVI	GNDVI = (R_750_ − R_540_ + R_570_)/(R_750_ + R_540_ − R_570_)	[66]
20	Simple Ratio	SR	SR = R_900_/R_680_	[67]
21	Red-Edge NDVI	RNDVI	RNDVI = (R_750_ − R_705_)/(R_750_ + R_705_)	[68]
22	Modified Triangular Vegetation Index	MTVI	MTVI = 1.2 × (1.2 × (R_800_ − R_550_) − 2.5 × (R_670_ − R_550_))	[59]
23	Triangular Vegetation Index	TVI	TVI = 0.5 × (120 × (R_761_ − R_581_) − 200(R_651_ − R_581_))	[69]
**Physiology**				
24	Fluorescence Ratio Index 1	FRI_1_	FRI1 = R_690_/R_630_	[70]
25	Fluorescence Ratio Index 2	FRI_2_	FRI2 = R_750_/R_800_	[71]
26	Fluorescence Ratio Index 3	FRI_3_	FRI3 = R_690_/R_600_	[72]
27	Fluorescence Ratio Index 4	FRI_4_	FRI4 = R_740_/R_800_	[72]
28	Fluorescence Curvature Index	FCI	FCI = R^2^_683_/(R_675_×R_691_)	[70]
29	Modified Red-Edge Simple Ratio Index	mRESR	mRESR = (R_750_ − R_445_)/(R_705_ + R_445_)	[73]
30	Normalized Pheophytization Index	NPQI	NPQI = (R_415_ − R_435_)/(R_415_ + R_435_)	[74]
31	Red-Edge Vegetation Stress Index 1	RVS1	RVS1 = ((R_651_ + R_750_)/2) − R_733_	[75]
32	Red-Edge Vegetation Stress Index 2	RVS2	RVS2 = ((R_651_ + R_750_)/2) − R_751_	[75]
**Water content**				
33	Water Index	WI	WI = R_900_/R_970_	[76]
34	Water Stress and Canopy Temperature	WSCT	WSCT = (R_970_ − R_850_)/(R_970_ + R_850_)	[77]

## Data Availability

Exclude this statement.
